# Differential β_2_-adrenergic receptor expression defines the phenotype of non-tumorigenic and malignant human breast cell lines

**DOI:** 10.18632/oncotarget.2460

**Published:** 2014-10-18

**Authors:** Lucía Gargiulo, Sabrina Copsel, Ezequiel M. Rivero, Céline Galés, Jean-Michel Sénard, Isabel A. Lüthy, Carlos Davio, Ariana Bruzzone

**Affiliations:** ^1^ Instituto de Biología y Medicina Experimental-CONICET. Vuelta de Obligado 2490. C1428ADN, CABA, Argentina; ^2^ Laboratorio de Farmacología de Receptores. Departamento de Farmacología. Facultad de Farmacia y Bioquímica. Universidad de Buenos Aires. Junin 956 (1113) CABA, Argentina; ^3^ Institut des Maladies Métaboliques et Cardiovasculaires. Institut National de la Santé et de la Recherche Médicale. U1048, Université Toulouse III Paul Sabatier, F-31432 Toulouse, France

**Keywords:** human breast cancer cells, non-tumorigenic breast cells, epinephrine, adrenergic receptors

## Abstract

Breast cancer is the most frequent malignancy in women. Several reports demonstrated that adrenergic receptors (ARs) are involved in breast cancer. Here we observed that epinephrine (Epi), an endogenous AR agonist, caused opposite effects in non-tumorigenic (MCF-10A and HBL-100) and tumor cells (MCF-7 and MDA-MB-231). Thus, Epi, in non-tumor breast cells, as well as isoproterenol (β-agonist), in all cell lines, maintained a benign phenotype, decreasing cell proliferation and migration, and stimulating cell adhesion. β-AR expression and cAMP levels were higher in MCF-10A than in MCF-7 cells. β_2_-AR knock-down caused a significant increase of cell proliferation and migration, and a decrease of cell adhesion both in basal and in Iso-stimulated conditions. Coincidently, β_2_-AR over-expression induced a significant decrease of cell proliferation and migration, and an increase of cell adhesion. Therefore, β_2_-AR is implied in cell phenotype and its agonists or antagonists could eventually complement cancer therapy.

## INTRODUCTION

Breast cancer is the most frequent malignancy in women worldwide, accounting for 23% of cancers in women, and the main cause of cancer death in women in both developing and developed countries. In USA, however, lung cancer has surpassed breast cancer mortality in women around 1990 [1, 2]. The molecular profiling of different subtypes of breast cancer greatly improved treatments and outcomes for patients [3].

Stress response is mediated primarily by the hypothalamic-pituitary-adrenocortical axis and the autonomic nervous system [4]. Epinephrine (Epi, also known as adrenaline) and Norepinephrine (NEpi, or noradrenaline) are classic neurotransmitters that mediate stress responses from the autonomic nervous system (a mechanism usually stated as “fight or flight” response). These catecholamines bind to three types of adrenergic receptors (ARs), subdivided in 9 different subtypes: α_1A_, α_1B_, α_1D_, α_2A_, α_2B_, α_2C_, β_1_, β_2_, β_3_ [5]. ARs belong to the G protein-coupled receptors (GPCRs), which regulate several physiological and pathological conditions. The stimulation of β_2_-AR leads to Gs-dependent adenylyl cyclase activation and cAMP production which activates protein kinase A (PKA) and exchange protein directly activated by cAMP (Epac). Also, desensitization of β_2_-AR is mediated by GPCR kinase-dependent phosphorylation, followed by the recruitment of β-arrestins, independent signal transducers [6].

The β-AR pathway has been implied in different processes of cancer initiation and progression [7]. It has been previously shown both by us and other researchers, that β-AR stimulation was associated to an inhibition of cancer cell proliferation and tumor growth [8–10]. However, β-blockers showed a similar inhibition of these parameters [11]. In addition, breast cancer cell motility is modified by adrenergic compounds in a cell-context manner, either enhancing or inhibiting this parameter [12, 13]. Our group has recently investigated the effect of the β-agonist isoproterenol (Iso) in non-tumorigenic human breast MCF-10A cells. Iso significantly diminished cell proliferation by a β_2_-AR/Gs/cAMP/PKA/Erk1/2 pathway and also caused a significant enhancement of cell adhesion mediated mainly by β_2_-AR/Gs/cAMP/Epac [14]. In the last decades, β-ARs have gained great importance in breast cancer treatment due to β-blockers, mainly propranolol, being used in the treatment of patients with very promising outputs [15, 16].

Altogether, these previous findings encouraged us to study the role of β-AR in cell proliferation, adhesion and migration, key processes of tumor progression. For this purpose, tumor (MCF-7 and MDA-MB-231) and non-tumorigenic (MCF-10A and HBL-100) human breast cell lines were used. Both tumor cell lines were selected because they represent opposite breast cancer phenotypes. MCF-7 as a luminal A model, that accounts for more than 30% of breast cancer patients, and MDA-MB-231, previously considered basal-like, but now reclassified within the claudin-low subtype, corresponds to around 10% of breast tumors [17]. Our study demonstrated for the first time that β_2_-AR expression is implicated in breast cell phenotype, suggesting that this receptor might be an important indicator of cell malignancy and consequently of tumor progression.

## RESULTS

**Figure 1:**
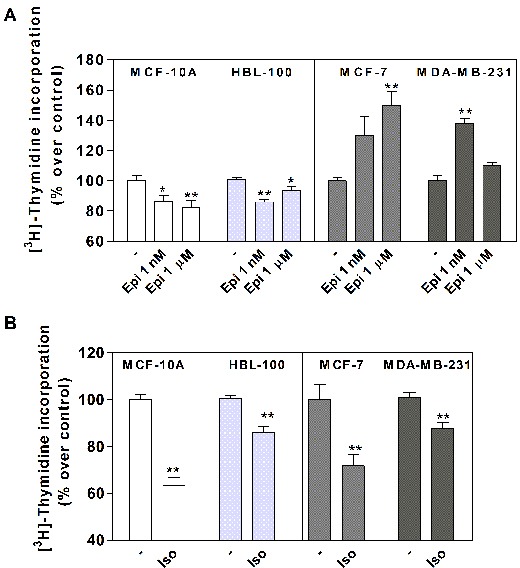
Effect of epinephrine (Epi) and isoproterenol (Iso) on MCF-10A, HBL-100, MCF-7 and MDA-MB-231 cell proliferation **(A)** Cells were stimulated or not (−) with 1 nM or 1 μM Epi or **(B)** 1 μM Iso and cell proliferation was evaluated by DNA synthesis using [^3^H]-Thymidine incorporation assay. The values are expressed as a percentage of the control in the absence of agonists. Data represent the mean ± s.e.m. of three independent experiments. Statistical significance was assessed using (**A**) ANOVA followed by a Dunnett’s test or (**B**) unpaired Student’s t-test.* p<0.05, **p<0.01.

As epinephrine (Epi) is the endogenous AR ligand that binds to all ARs, we assessed its action on different parameters linked to breast cancer progression, such as proliferation, adhesion and migration. Cell proliferation was measured by [^3^H]-Thymidine incorporation in non-tumorigenic MCF-10A and HBL-100 cell lines, and in breast tumor cell lines MCF-7 and MDA-MB-231 (Figure 1A). Both Epi concentrations (1 nM and 1 μM) caused a significant inhibition of cell proliferation in the non-tumorigenic cells, whereas it significantly increased cell proliferation at a concentration of 1 μM in the cancer cells. We previously demonstrated in several human breast cell lines that Epi induced proliferation through α_2_-AR stimulation [9, 18]. On the other hand, Isoproterenol (Iso), a β-AR agonist, significantly decreased cell proliferation in every cell line (*p<0.01*, Figure 1B).

**Figure 2:**
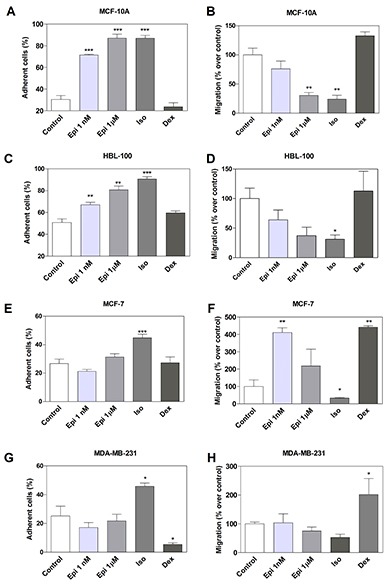
Effect of adrenergic compounds on cell adhesion and migration in MCF-10A, HBL-100, MCF-7 and MDA-MB-231 cells **(A, C, E and G)** Cells were stimulated or not (Control) for 15 min with 1 nM or 1 μM epinefrine (Epi), 1 μM isoproterenol (Iso, β-AR agonist) and 1 μM dexmedetomidine (Dex, α_2_-AR agonist) and treated with the specific cell detachment buffer as described in the *Materials and Methods* section. Results are expressed as the percentage of cell number remaining adherent to the plastic dishes following specific cell detachment treatment. **(B, D, F and H**) Cell migration was measured by transwell assay treated during 16 hs with the same drugs as before. Data represent the mean ± s.e.m. of three independent experiments. Statistical significance was assessed using ANOVA followed by a Dunnett’s test.* p<0.05, **p<0.01, ***p<0.001.

The effect of adrenergic compounds on cell adhesion was next evaluated by quantifying the percentage of cells remaining adherent to the plastic dishes following a specific cell detachment treatment as described in *Materials and Methods*. In non-tumorigenic breast cells MCF-10A, treatment with Epi resulted in a large and significant increase in the proportion of adhesive cells (Figure 2A). As Epi binds to α_2_ and to β-AR, which have been described in both tumor and non-tumorigenic breast cell lines [14, 18, 19], an agonist of each receptor type was also used in order to identify through which receptor Epi exerts this action. The significant increase of cell adhesion caused by Epi in non-tumorigenic cells at both concentrations was mimicked by the treatment with the β-adrenergic agonist Iso (*p*<0.01), but not by the α_2_-adrenergic agonist Dexmedetomidine (Dex) (Figure 2A). Similar results were observed in the non-tumorigenic breast cell line HBL-100 (Figure 2C). Coincidently with this finding, in MCF-10A cells, 1 μM Epi and Iso significantly inhibited cell migration (Figure 2B). In HBL-100 cells, Iso caused a significant decrease of cell migration, although no significant differences were observed with Epi treatment. In addition, Dex had no effect in either cell line on attachment or migration (Figure 2A-D).

In MCF-7 cells, Iso caused a moderate though significant increase in cell adhesion (*p*<0.001, Figure 2E), with a coincident inhibition of cell migration (*p*<0.05, Figure 2F). However, even if no effect on cell adhesion was evident in the presence of Epi or Dex in MCF-7 cells (Figure 2E), the lower concentration of Epi as well as Dex significantly increased cell migration (Figure 2F). In MDA-MB-231 cells the effects of Iso and Dex were opposite, and therefore, Epi which binds to both kinds of receptor showed no differences in cell adhesion (Figure 2G). In these cells, cell migration was significantly increased by Dex treatment (Figure 2H).

**Figure 3:**
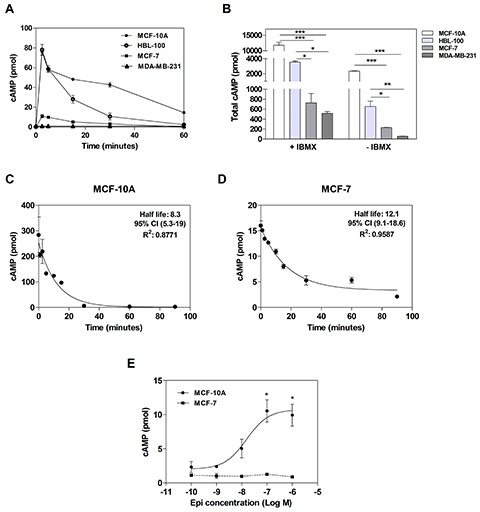
cAMP levels in tumor and non-tumorigenic breast cells **(A)** Time course of intracellular cAMP production in MCF-10A, HBL-100, MCF-7 and MDA-MB-231 cells in the absence of IBMX. **(B)** Comparison of the total production of cAMP (area under the curve obtained in A) after 1 μM isoproterenol stimulation in the presence (+) or absence (−) of 1 mM IBMX. **(C and D)** Desensitization of β-AR in MCF-10A cells **(C)** or MCF-7 cells **(D)**. **(E)** cAMP concentration-response curves to Epinephrine (Epi) in MCF-10A and MCF-7 cells with 1 mM IBMX. Data represent the mean ± s.e.m. of three independent experiments. Statistical significance was assessed using ANOVA followed by Bonferroni (B) or Dunnett’s test (E).* p<0.05, **p<0.01, ***p<0.001.

So far, we demonstrated an inhibition of cell proliferation and migration and an increase of adhesion when the non-tumor and tumor cells were incubated with Iso. Due to the previously mentioned importance of β-AR in cancer biology and our results, we next focused particularly on this receptor. Since the stimulation of β-AR causes an increase in cAMP levels, we determined its levels after 1 μM Iso treatment. Intracellular cAMP levels were studied in all cell lines in the presence or absence of the phosphodiesterase (PDE) inhibitor 3-isobutyl-methylxantine (IBMX) (Figure 3A and B). Without IBMX, the peak concentration was coincident at 2.5 minutes. However, the non-tumorigenic breast cell lines, MCF-10A and HBL-100, exhibited higher levels of cAMP than the cancer MCF-7 and MDA-MB-231 cells (Figure 3A). When the total area under the curves was evaluated with or without IBMX, non-tumorigenic cells exhibited higher cAMP levels than tumor cell lines (Figure 3B). As MCF-10A and MCF-7 cells are suitable models of non-tumorigenic and tumor cell lines respectively, and they showed the highest cAMP levels within each group, they were chosen for further experiments.

In order to explain the results shown in Figure 1 and 2, where Epi acts differentially in non-tumor *vs* tumor breast cell lines, we determined the number of β-AR in MCF-10A and MCF-7 cells by binding assays. The β-AR levels were higher in MCF-10A than in MCF-7 cells (MCF-10A: 132 ± 21×10^3^ *vs* MCF-7: 80 ± 5.5×10^3^ sites/cell, *p*<0.05). Furthermore, we previously reported the number of α_2_-binding sites/cell was higher in MCF-7 than in MCF-10A (MCF-7: 103 ± 23×10^3^ *vs* MCF-10A: 19 ± 3.5×10^3^ sites/cell) [18].

To evaluate β-AR desensitization, we studied the ability of this receptor to continue producing cAMP after a stimulus. Cells were incubated at different times in the presence of 1μM Iso (without IBMX) washed with cold PBS and re-stimulated for 10 minutes (with IBMX). cAMP levels were then measured [20]. No differences were found in desensitization between both cell lines (Figures 3C and D). These results confirm that the higher cAMP levels observed in MCF-10A compared with MCF-7 cells (Figure 3A) were due to the differences in β-AR expression and not to a differential desensitization rate of this receptor.

In order to evaluate the effect of Epi, the natural agonist of AR, on cAMP production, concentration-response curves were also performed. The incubation of MCF-10A cells with increasing concentrations of Epi elicited a marked enhancement of cAMP concentrations (in the presence of IBMX) while the incubation of MCF-7 cells did not change cAMP levels (Figure 3E). This last result on cAMP production could be explained by the high expression of α_2_-AR in this cell line, which classically couple to G_o/i_ protein, inactivating adenylyl cyclase [18].

**Figure 4:**
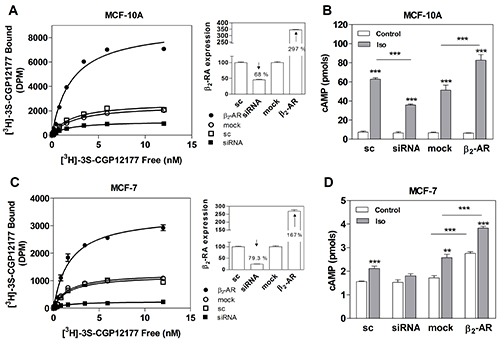
β_2_-AR overexpression and knock-down in MCF-10A and MCF-7 cells **(A)** Quantification of β_2_-AR in MCF-10A and **(C)** MCF-7 cells transfected with scrambled siRNA (sc), β_2-_AR-targeted pooled siRNA (siRNA), pcDNA3.1 (mock) or the plasmid codifying for the β_2_-AR. Panels **A** and **C** depict the saturation analysis performed with the β-AR radioligand [^3^H]-GCP 12177. The results are expressed as the percentage of the scrambled or the mock in whole cells at 4 °C. The modification of the expression of β-AR in the cells is shown in insets as a percentage of the sc or mock. (**B**) Total cAMP production in MCF-10A cells or (**D**) MCF-7 cells transfected with sc, siRNA, mock or β_2_-AR and incubated or not (control) with 1 μM Isoproterenol (Iso). Data represent the mean ± s.e.m. of two independent experiments. Statistical significance was assessed using ANOVA followed by a Bonferroni test. *** p<0.001.

Since the β_2_-AR is the most expressed β-AR subtype in breast cell lines, including MCF-10A and MCF-7 cells [14, 19, 21, 22], we modified the expression levels of this receptor and evaluated its effect on proliferation, adhesion and migration. Cells were transfected either with a small interference RNA (siRNA) for knocking down β_2_-AR expression [23], or with a human β_2_-AR plasmid [24] for over-expressing it. As controls, both cell lines were also transfected with a scrambled siRNA (sc) or an empty vector (mock). β_2_-AR concentrations were analysed by binding assays (Figure 4A for MCF-10A and 4C for MCF-7) and receptor functionality was studied by measuring cAMP levels. As shown in Figure 4B, when modifying β_2_-AR levels in MCF-10A cells, cAMP basal concentrations did not change. However, the Iso-stimulated concentrations of cAMP were highly dependent on the β_2_-AR expression levels (Figure 4B). In MCF-7, β_2_-AR knock-down abrogated Iso cAMP stimulation (Figure 4D). Moreover, β_2_-AR over-expression caused a significant increase of cAMP levels in both basal and Iso-stimulated conditions, showing the important basal activity of the receptor.

**Figure 5:**
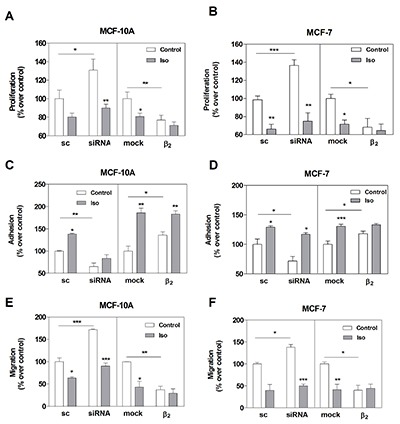
Effect of β_2_-AR expression on cell proliferation, adhesion and cell migration in MCF-10A and MCF-7 cell lines Cells were transfected with scrambled siRNA (sc), β_2_**-**AR-targeted pooled siRNA (siRNA), pcDNA3.1 (mock) or the plasmid codifying for the β_2_-AR. The results are expressed as the percentage of the sc or mock. Panels **A** and **B**: cells were stimulated or not (Control) with 1 μM Isoproterenol (Iso) and cell proliferation was evaluated by automatic cell-counting. Panels **C** and **D**: cells were stimulated or not (Control) for 15 min with 1 μM Iso and treated with the specific cell detachment buffer as described in *Materials and Methods*. Results are expressed as the percentage of cell number remaining adherent to the plastic dishes following specific cell detachment treatment. Panels **E** and **F:** cell migration was measured by transwell assay treated or not (Control) with 1 μM Iso during 16 hs. Data represent the mean ± s.e.m. of three independent experiments. Statistical significance was assessed using ANOVA followed by a Bonferroni test.* p<0.05, **p<0.01, ***<p0.001.

When we evaluated parameters related to tumor phenotype in MCF-10A and MCF-7 cells, we found that β_2_-AR knock-down caused a significant increase in cell proliferation and migration, and a decrease in cell adhesion not only in basal but also in Iso-stimulated conditions (Figure 5). In line with this, β_2_-AR over-expression induced a significant decrease in cell proliferation and migration, and an increase in cell adhesion (Figure 5). Since β_2_-AR over-expression mainly affected basal conditions, no differences were observed when cells were stimulated with Iso.

## DISCUSSION

Catecholamines, epinephrine (Epi) and norepinephrine (NEpi), are released during both acute and chronic stress. Their functions are mediated through binding to adrenergic receptors (AR). Classically, these receptors were associated with the regulation of body homeostasis in health and disease, including cardiac and neurologic function. In the last decade, several reports reinforced the idea that ARs have an implication in cancer biology [25, 26]. It was shown that these receptors are involved in breast cancer progression. In particular, more than two decades ago, β-ARs were described in breast cancer cells in human and in experimental models [19, 27]. Since then, controversial results have been found with respect to agonist action on several parameters related to breast tumor progression, as reviewed in [28]. Numerous studies have shown an inhibition of cell proliferation and cell migration by β-AR agonists [8, 9, 13, 29]. Interestingly, β-blockers, commonly used as antihypertension drugs, have also been identified as decreasing cell proliferation and cell migration in estrogen receptor positive and negative breast cancer cells [14, 15], but also in other tumor types, including lung, pancreas, prostate, colon, stomach and ovarian cancer [30]. These controversial actions between β-agonists and β-blockers could be explained by the presence of polymorphism in the β_2_-AR gene or due to the recently discovery that some β-blockers have β-arrestin–biased agonism activity [30, 31]. Recently, an α_1_-blocker, also used as an antihypertension drug, inhibited tumor growth by reducing vascularization in an ovarian cancer model [32].

Due to the relevance of β-AR expression and activation in cancer, we first aimed on studying the role of Epi in tumor (MCF-7 and MDA-MB-231) and non-tumorigenic (MCF-10A and HBL-100) human breast cell lines. The MCF-7 cell line was obtained from a pleural effusion of a human breast cancer [33] and is a paradigm of luminal A cells [17]. On the other hand, MDA-MB-231 cell line, also derived from a pleural effusion [34], represents a more aggressive breast tumor. This triple-negative cell line was classified as claudin-low [17]. Both cell lines are well accepted breast cancer *in vitro* models. They were selected not only because they represent opposite types of tumor, but also because MCF-7 is a hormone dependent model, and MDA-MB-231 is an independent one. On the other hand, the MCF-10A cell line arose from a breast cyst of a premenopausal woman [35] and has been considered triple negative [36]. HBL-100, not tumorigenic at low passages, was originally isolated from the milk of an apparently healthy woman [37] and has been classified as claudin-low [17]. Both cell lines are well-accepted models of a non-tumor breast.

In this work, Epi was chosen since it preferentially binds to β_2_-AR (and NEpi to β_1_-AR) and this receptor is the main β subtype expressed in both tumor and non-tumorigenic breast cells [14, 19, 38, 39]. Epi plasma concentration is extremely variable when comparing data from the literature. However, its basal concentration is around 1 nM which can rise 10-fold within seconds during acute stress [7, 40]. For this reason, we decided to evaluate the effect of two different Epi concentrations (1 nM and 1μM). Interestingly, when investigating the effect of Epi in cell proliferation, adhesion and migration, the action was opposite in the non-tumorigenic and the tumor cells. In MCF-10A and HBL-100, Epi tended to maintain a benign phenotype, decreasing cell proliferation and migration, and stimulating cell adhesion. The same behavior was observed when cells were stimulated with the β-agonist, Iso. On the other hand, Epi induced cell proliferation and cell migration on MCF-7 tumor cells. This effect on cell proliferation is mediated by α_2_-AR, as we have previously described [18]. Moreover, in this study dexmedetomidine, an α_2_-agonist, also induced a decrease in MDA-MB-231 cell adhesion, and an increase in cell migration in both tumor cell lines. In particular, in MCF-7, when increasing Epi concentration to 1 μM, no significant differences were observed in terms of cell migration. This could be due to the fact that at nM concentration, actions are mediated preferentially by α_2_-AR, and at μM concentration the β-AR effect compensates for the α_2_-AR effect. This dual Epi action, was previously described in the proliferation of breast cancer cell lines [9]. In conclusion, Epi response could then be interpreted as mainly mediated by β-AR in non-tumorigenic cells and by α_2_-AR in tumor cells.

ARs are widely over-expressed in numerous types of human breast tumor. In particular, an enhanced β_2_-AR expression was found in the luminal subtype with low clinical stage, low proliferation and good prognosis [41]. In line with this, the present study shows that the non-tumorigenic MCF-10A cell line express higher levels of β-AR than the tumor MCF-7 cell line. On the other hand, Powe et al. described that α_2C_-AR are highly expressed in breast tumors with a more aggressive phenotype, which is in line with our previous observation where α_2_-AR binding sites were higher in tumor breast cell lines in comparison with the non-tumorigenic cells [18].

cAMP production is the classical second messenger involved in the β-AR pathway. Therefore, we determined its levels in the non-tumorigenic and tumor breast cell lines. cAMP levels were higher in the non-tumorigenic MCF-10A and HBL-100, intermediate in MCF-7 and lowest in MDA-MB-231. These findings suggest that there seems to be a progression toward less cAMP production when the cells are more malignant. This is in line with Bodwin et al, who demonstrated, even before breast cancer β-AR description, that cAMP inhibits mammary tumor growth [42]. MDA-MB-231 cell line was described as expressing high levels of β_2_-AR [22]. However, as previously mentioned, low cAMP levels were detected. Therefore, in this cell line, the production of this second messenger does not seem to directly correlate with β_2_-AR concentration. Indeed, it is well known that the activity of GPCRs results from a coordinated balance among diverse mechanisms that govern receptor signaling at the different levels of signal propagation. Thus, our findings in the MDA-MB-231 cells could be explained by an imbalance in protein levels involved in the β-signaling pathway or an impaired coupling of the β_2_-AR/G protein/adenylyl cyclase. Further detailed studies are necessary to determine the cause of these low cAMP levels detected in MDA-MB-231 cells.

cAMP concentration is the result of its production, which is diminished by desensitization, its degradation by phosphodiesterases (PDEs) and/or the efflux by multidrug resistance-associated proteins. In particular, desensitization has been extensively studied for the β_2_-AR. It was described as a physiologically mechanism triggered by constant or repeated stimuli, which protects the cell from both short- and long-term receptor overstimulation [43]. The results of cAMP production showed that MCF-10A and MCF-7 produced higher levels of this cyclic nucleotide within non-tumorigenic or tumor cell categories. Thus, these two cell lines were chosen in order to evaluate the role of β_2_-AR on the tumor phenotype. Since MCF-10A showed higher β_2_-AR and cAMP levels than MCF-7, we then focused on studying β-desensitization, a process that directly associates cAMP levels to functional β-AR in cell membrane. No differences were observed in the capacity of the endogenous receptor to continue being stimulated, assessed as a measure of all these processes summarized as desensitization. The time-course of cAMP production showed a parallel curve, suggesting that the degradation should also be similar in both cell lines. Therefore the higher cAMP concentration in MCF-10A cells seems to be due to a higher production in this cell line, which should be at least partially due to the higher β_2_-AR concentration observed. However, a better coupling of the receptor to the G protein and adenylyl cyclase cannot be discarded.

To further investigate the effect of β_2_-AR concentration in the breast cell phenotype, this receptor was successfully over-expressed or knocked-down in MCF-10A and MCF-7 cell lines, as confirmed by the binding assays. A strong effect on cell proliferation, adhesion and migration was observed in both cell lines when the β_2_-AR was silenced or over-expressed. These findings strengthen β_2_-AR relevance in cell processes implied in tumor progression. When MCF-10A cells were stimulated with the β-AR agonist, cAMP production highly correlated with cell proliferation, adhesion and migration. However, the basal levels were not modified by over-expressing or silencing β_2_-AR. This could be explained by cAMP compartmentalization. In fact, it has recently been described that the proximity of PDEs to the site of cAMP synthesis restricts its dissemination through the cell [44]. Moreover, the stimulation of β_1_-AR induces a cAMP gradient that propagates throughout the cells, while localized β_2_-AR stimulation does not trigger cAMP diffusion [45]. On the other hand, classical approaches to measuring total cAMP production with poor spatiotemporal resolution most probably miss cAMP synthesis in discrete cell compartments. All in all, in MCF-10A cells slight changes of cAMP levels in specific cell compartments could translate into important changes in biological actions. In MCF-7 cells however, the over-expression of the receptor enhanced both the basal and the stimulated cAMP levels, showing an important basal activity of the receptor in these cells. Unliganded β_2_-AR was described coupling to Gs, explaining the basal activity [46].

Experiments investigating the possibility of using α_2_ and β-agonists and antagonists for inhibiting cell migration and invasion and enhancing cell adhesion are advanced in our laboratory, as well as the signaling pathways involved in these processes.

In conclusion, the present study demonstrates for the first time that not only activation, but also β_2_-AR expression regulates breast tumor cell phenotype, modifying proliferation, adhesion and cell migration. Thus, the level of β_2_-AR expression and its functionality could be important indicators of cell malignancy and consequently of tumor progression. Additionally, as similar results were obtained in two different and opposite tumor models, the role of β_2_-AR in breast cancer might be independent of the breast cancer molecular subtype reinforcing its relevance in clinical translation. Future studies using patient and normal breast samples are necessary to gain greater insight for the implication of β_2_-AR on breast cancer.

## MATERIALS AND METHODS

### Drugs and reagents

Methyl-[^3^H]-thymidine (NET 027E; specific activity: 20 Ci/mmol) was from Dupont-New England Nuclear (Boston, MA, USA). [^3^H]-cAMP (31 Ci/mmol) and [^3^H]-CGP12177 (30 Ci/mmol) were from Perkin Elmer Life Sciences (Waltham, MA, USA). Insulin, epidermal growth factor (EGF), hydrocortisone, (−)epinephrine, isoproterenol, 3-isobutyl-methylxantine, were from Sigma-Aldrich (St Louis, MO, USA). Dexmedetomidine was purchased from Abbot (Buenos Aires, Argentine). Culture medium, fetal calf serum (FCS), Lipofectamine 2000, siRNA (ADRB2 Stealth Select RNAi™, HSS100258, HSS100259, y HSS100260) and other products for cell culture were from Invitrogen (Carlsbad, CA, USA). FuGENE® was purchased from Promega (Madison, WI, USA) and the transwell inserts were BD-Falcon (San Jose, CA, USA).

### Cell culture and transfection

MCF-10A, MCF-7, MDA-MB-231 and HBL-100 cells lines were obtained from the American Type Culture Collection (ATCC, Manassas, VA, USA) and were cultured as already described [18] with Hepes-buffered DMEM/Ham F12 culture medium (basal medium) supplemented with antibiotics (100 μg/mL streptomycin, 100 IU/mL penicillin), 10% FCS and 2 μg/mL human insulin (complete medium). For MCF-10A cells the medium was also supplemented with 20 ng/mL EGF and 0.1 μM hydrocortisone. Cells were sub-cultured once weekly by trypsinization (0.25% trypsin–0.025% EDTA) and the medium was changed every second or third day.

For transfection, 5×10^5^ cells/well were seeded in 24 well-plates in complete medium. The receptors were knocked down with small interfering RNA (siRNA) as previously described [23]: 10 nM of three different sequences of siRNA targeting β_2_-AR, the mixture of them or 10 nM non-targeting scrambled siRNA (sc), used as a control. The β_2_-AR was overexpressed by transfecting 1.5 μg per well of a plasmid already described [24]. The control was the empty vector pcDNA3.1 (mock). The plasmids and siRNAs were prepared in OPTIMEM medium. For MCF-10A cells, FuGENE was used for 18 h [14], whereas for MCF-7 cells Lipofectamine 2000 was used for 6 h. After transfection, the medium was removed and replaced by fresh complete medium. Proliferation, adhesion and migration assays were performed between 48 and 72 h post-transfection. Receptor silencing or over-expression was evaluated by binding assays.

### Cell proliferation

Cells were seeded in 96-well plates (5×10^3^ cells/well) and grown during 24 h in complete medium. After 24 h the medium was changed to 2% charcoal-stripped FCS and the cells were treated with the compounds described. The treatment was repeated an additional day with the addition of 0.2 μCi methyl-[^3^H]-thymidine/well. Cells were harvested in a Nunc Cell Harvester 8 and radioactive nuclei retained into the glass fiber filters were counted in a liquid scintillation counter. The results are expressed as percentage of the control incubated in the absence of any compound. Proliferation response in transfected cells was evaluated by automatic cell-counting (Beckman Coulter Z1 Cell-counter).

### Measurement of cell adhesion

Cells were seeded in 12-well plates (1.5×10^5^ cells/well) for 24 h, the medium was removed, and cells were treated or not with different adrenergic compounds during 15 min in basal medium. The medium was removed and the cells were incubated in Mg^2+^/Ca^2+^-free PBS containing 0.5 mM EDTA and 0.25% trypsin as previously described in constant agitation at room temperature during 15 min for MCF-10A cells [14], or 5 min for MCF-7, MDA-MB-231 and HBL-100 cells. Cells that resisted the treatment and remained adherent to the plastic were harvested following an additional 30 min incubation in Mg^2+^/Ca^2+^-free PBS containing 2.5 mM EDTA and 1.25% trypsin and counted (attached cells) using a cell counter. The percentage of adherent cells was calculated as follows: attached cells × 100/ (attached cells + detached cells).

### Migration assay

Cell migration was evaluated using transwell inserts with 8 μm pore. 2×10^4^ cells were seeded in the upper compartment, onto the porous membrane, and were allowed to adhere during 4 h in complete medium. Afterwards, the medium was carefully removed and changed for a basal medium and stimulated with the adrenergic compounds to test. After 16 h, the medium was removed and the cells fixed and stained with 0.05% crystal violet in methanol during 10 min. Non-migrated cells were removed from the top of the membrane using a cotton swab. The total amount of migrated cells was counted on an inverted microscope.

### Radioligand binding assay

β-AR quantification was performed by binding analysis in whole cells at 4 °C to avoid ligand internalization, as described [23]. 7×10^4^ cells/well were seeded in 48 well plates. The number of binding sites was evaluated using the antagonist [^3^H]-GCP12177 in the presence or absence of 100 μM Iso at 4 °C [23]. The incubation was stopped by dilution with 3 ml of ice-cold 50 mM Tris-HCl, pH 7.4. After three washes with 3 ml of ice-cold buffer, the bound fraction was collected in 200 μl of ethanol.

### cAMP cell content quantification

cAMP content was quantified using a competitive radio-binding assay for PKA using [^3^H]-cAMP, as previously described [47]. Cells were seeded in 24-well plates (1.7×10^5^ cells/well) in complete medium. After 24 h, the medium was removed and cells were incubated in RPMI medium without phenol red at 37 °C with different adrenergic compounds during the indicated times with or without 1mM of the PDE inhibitor 3-isobutyl-methylxantine (IBMX) at 37 °C. Ethanol was added to stop the reaction. The extracts were centrifuged for 3 min at 3000×*g* and the recovered supernatant was evaporated and then resuspended in 50 mM Tris-HCl, pH 7.4, 0.1% BSA for cAMP quantification. Standard curves were performed with 8 different cAMP concentrations, ranging from 0.1 to 90 pmols. The data shown are the result of duplicates from at least three independent experiments.

### Statistical analysis

The experiments were repeated at least twice with identical results. The analyses performed were Student’s t test or ANOVA followed by Bonferroni or Dunnet’s test in the case of multiple comparison. The differences were considered significant when p<0,05 [48]. Binding data, sigmoidal dose-response, and desensitization fittings were performed with GraphPad Prism 5.00 for Windows (GraphPad Software, San Diego, CA).
